# Laboratory evaluation of the optical properties of two extended-depth-of-focus intraocular lenses

**DOI:** 10.1186/s12886-020-1332-6

**Published:** 2020-02-14

**Authors:** Sue Hey Chae, Hyeck Soo Son, Ramin Khoramnia, Kyung Heon Lee, Chul Young Choi

**Affiliations:** 1 Sungmo Eye Hospital, Busan, Republic of Korea; 2 The David J. Apple International Laboratory for Ocular Pathology and International Vision Correction Research Centre (IVCRC), Department of Ophthalmology, University of Heidelberg, Heidelberg, Germany; 3 Department of Ophthalmology, Kangbuk Samsung Hospital, Sungkyunkwan University School of Medicine, Pyeong-Dong, Jongno-Gu, Seoul, Republic of Korea

**Keywords:** Optical quality, Intraocular lenses, Extended-depth-of-focus, Pupil size

## Abstract

**Background:**

To experimentally compare the optical performance of two different Extended-Depth-of-Focus (EDOF) intraocular lenses (IOLs) using a standardized optical bench set-up.

**Methods:**

In this experimental study, following IOLs were assessed: the TECNIS® Symfony ZXR00 (Johnson&Johnson, Santa Ana, USA) and the AT LARA 829MP (Carl Zeiss Meditec, Jena, Germany) IOLs. The through-focus modulation transfer function (MTF) values were measured at a spatial frequency of 50 lp/mm and at aperture sizes of 2, 3, and 4.5 mm. Each IOL was measured while centered using ISO 11979-2 Model 1 (aberration-free) and Model 2 (+ 0.28 μm spherical aberration) corneas. United States Air Force (USAF) target images were also recorded for a qualitative evaluation.

**Results:**

At 2 mm pupil with ISO1 cornea, the primary and secondary foci of both IOLs appeared to merge, providing an elongated depth of focus. At 3 and 4.5 mm pupil sizes, the through-focus MTF curves of both IOLs showed a bifocal-like V-pattern. While the Symfony IOL showed an overall superior MTF values when measured with the ISO2 cornea, the opposite propensity could be observed with the AT LARA IOL. This optical behavior could be qualitatively confirmed by the USAF target images.

**Conclusions:**

Although the two EDOF IOLs share similarities in their optical properties, the main difference lies in their optical design and performance with respect to spherical aberration. Such characteristics should be taken into account during IOL and patient selection.

## Background

Presbyopia-correcting IOLs can be largely categorized depending on the optical principle and the number of foci generated [1–3]. Most IOLs use a diffractive-refractive optic, while others utilize different optical designs to provide multifocality [4, 5]. The initial presbyopia-correcting IOLs were bifocal, providing functional vision at far and near distances [3, 6]. The subsequent development of trifocal IOLs allowed visual restoration in intermediate distance as well, enabling patients to use computers or tablets without spectacles [7–9].

Recently, so-called EDOF IOLs were introduced, which are claimed to restore vision across multiple distances by generating a broad dioptric range rather than a fixed number of foci [10, 11]. The TECNIS® Symfony ZXR00 and AT LARA 829MP are examples of such EDOF IOLs that can allow visual restoration by increasing the depth of focus. Although they both have a diffractive-refractive optic and share some similarities in their optical properties, they do differ in the optical design. Therefore, the aim of this laboratory study was to evaluate their optical behavior with respect to spherical aberration and pupil size using both aberration-free and + 0.28 μm spherical aberration model corneas.

## Methods

### Intraocular lenses

The following lenses were examined: the EDOF TECNIS® Symfony ZXR00 (Johnson&Johnson, Santa Ana, CA, USA) and the EDOF AT LARA® 829MP (Carl Zeiss Meditec, Jena, Germany) IOLs. Table 1 summarizes the main characteristics of the two IOLs. The Symfony IOL has a wavefront-designed, aberration-correcting, aspheric anterior surface and a fully diffractive, achromatic echelette posterior surface. The AT LARA IOL in contrast, is aberration-neutral and has a fully diffractive anterior surface with a smooth microphase design. For the purpose of this study, the two IOLs shared the same base dioptric power of + 20.0 D.

**Table 1:** Overview of the optical properties of the studied intraocular lenses

IOL	Symfony	AT LARA
Optic design	Aspheric, diffractive-refractive echelette feature	Aspheric, diffractive-refractive SMP (smooth microphase) design
Material	UV blocking hydrophobic acrylic	UV blocking, Hydrophilic acrylate (25% water content) with hydrophobic surface properties
Body design	Single-piece/C-loop, square edge	Single-piece, plate-haptic, square edge
Optical/total diameter (mm)	6/13	6/11
Dioptric power (D)	20.00	20.00
Spherical aberration (μm)	−0.27 μm	0.00 μm
Diffractive Steps (n)	9	15
Refractive Index	1.47	1.46
Abbe Number	55	56.5

### Optical quality measurement

The PMTF optical bench (Lambda-X, software version 2.11.2) is a dedicated optical set-up used for the image quality assessment of IOLs. It complies with the requirements of the ISO Standards 11,979– 2[12] and 11,979– 9[13] and allows the modulation transfer function (MTF) measurements at different apertures, spatial frequencies (through-frequency curve), and focal planes (through-focus curve). Its measurement principle is based on a patented quantitative deflectometry technique using deviation of light beams. The source wavelength is 545 nm. To assess the optical behavior of the IOLs, two different model corneas were used, as described in the ISO 11979-2: the ISO1 aberration-free model cornea and the ISO2 model cornea with an + 0.28 μm spherical aberration. The IOL under test is placed in an 11.0 mm diameter IOL holder and inserted into a wet cell filled with balanced salt solution. The anterior side of the IOL faces the incident light rays. The holder guarantees a tilt-free orientation of the IOL during measurement, which can be performed multiple times at various apertures without having to remove the IOL from the holder. Thus, all measurements were performed with the same IOL alignment.

The MTF values were recorded at different spatial frequencies and defocused planes to achieve through-focus curves for varying pupil apertures. The MTF values were measured at a spatial frequency of 50 lp/mm and at apertures sizes of 2, 3, and 4.5 mm. The United States Air Force (USAF) 1951 resolution target images were also recorded to qualitatively compare the optical performance. For each IOL model, five individual lenses were measured five times for each aperture size, yielding a total of 25 measurements per model. The overall mean and standard deviation values of the 25 individual measurements were then calculated for each IOL model.

### Statistical analysis

A nonparametric Mann-Whitney U Test was performed with the MedCalc statistical software for Windows (Version 15; MedCalc Software, Ostend, Belgium) for statistical analysis. A *p*-value of 0.05 or less was considered as statistically significant.

## Results

**Fig. 1:**
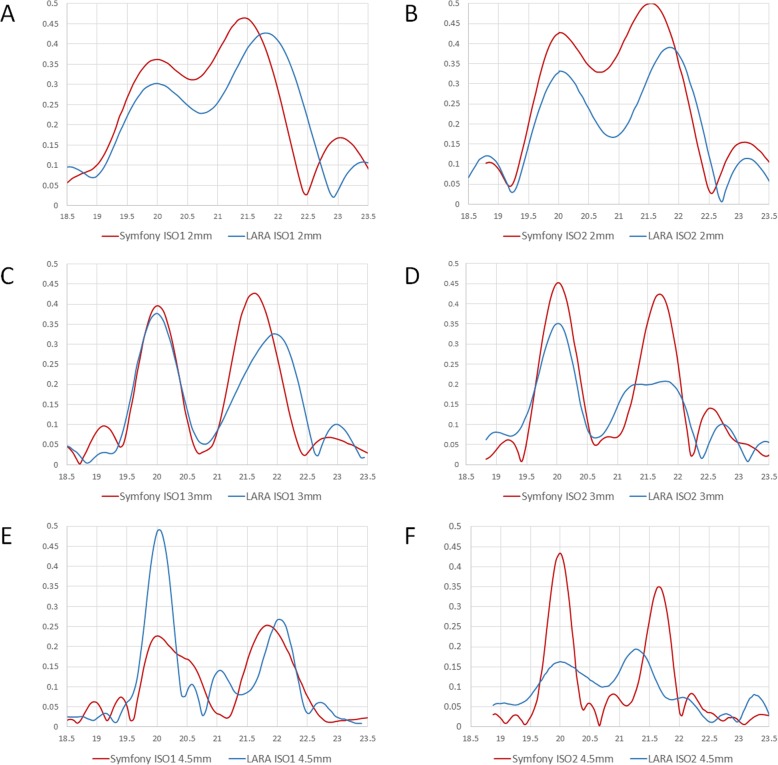
Through-focus curves at 50 lp/mm and at pupil sizes of 2, 3, and 4.5 mm, as measured with ISO1 and 2 model corneas

Figure 1 shows the through-focus MTF measurements of the two IOLs at 50 lp/mm and at pupil sizes of 2, 3, and 4.5 mm, with two different model corneas. As the ISO standard allows an IOL power tolerance of ±0.40 D for an official IOL power of + 20.0 D, the primary peak of IOLs were not always exactly at + 20.0 D. Therefore, we transposed the primary peaks to + 20.0 D for better comparison of the IOL’s depth of focus.

### Through-focus MTF with ISO1 model cornea at 2 mm pupil size

For primary focus, the mean MTF values were 0.361 and 0.311 for Symfony and AT LARA IOLs, respectively (*p* < 0.05). For secondary focus, the mean MTF values were 0.431 and 0.442 for Symfony and AT LARA IOLs, respectively, and this difference was not statistically significant (*p* = 0.31). The secondary focus of the AT LARA IOL was slightly higher (+ 1.85 D) than the former (+ 1.41 D).

### Through-focus MTF with ISO2 model cornea at 2 mm pupil size

For primary focus, the mean MTF values were 0.410 and 0.339 for Symfony and AT LARA IOLs, respectively (*p* < 0.05). For secondary focus, the mean MTF values were 0.424 and 0.380 for Symfony and AT LARA IOLs, respectively, and this difference was also statistically significant (*p* < 0.05). The secondary focus was measured at + 1.57 D for the Symfony IOL and at + 1.85 D for the AT LARA IOL.

### Through-focus MTF with ISO1 model cornea at 3 mm pupil size

For primary focus, the mean MTF values were 0.387 and 0.365 for Symfony and AT LARA IOLs, respectively (*p* < 0.05). For secondary focus, the mean MTF values were 0.416 and 0.323 for Symfony and AT LARA IOLs, respectively (*p* < 0.05). The secondary focus was measured at + 1.59 D for the Symfony IOL and at + 1.87 D for the AT LARA IOL.

### Through-focus MTF with ISO2 model cornea at 3 mm pupil size

For primary focus, the mean MTF values were 0.418 and 0.351 for Symfony and AT LARA IOLs, respectively (*p* < 0.05). For secondary focus, the mean MTF values were 0.395 and 0.209 for Symfony and AT LARA IOLs, respectively (*p* < 0.05). The secondary focus was measured at + 1.65 D for both Symfony and AT LARA IOLs.

### Through-focus MTF with ISO1 model cornea at 4.5 mm pupil size

For primary focus, the mean MTF values were 0.225 and 0.487 for Symfony and AT LARA IOLs, respectively (*p* < 0.05). For secondary focus, the mean MTF values were 0.252 and 0.257 for Symfony and AT LARA IOLs, respectively, and the difference was not statistically significant (*p* = 0.31). The secondary focus was measured at + 1.85 D for the Symfony IOL and at + 1.96 D for the AT LARA IOL.

### Through-focus MTF with ISO2 model cornea at 4.5 mm pupil size

For primary focus, the mean MTF values were 0.437 and 0.169 for Symfony and AT LARA IOLs, respectively (*p* < 0.05). For secondary focus, the mean MTF values were 0.344 and 0.187 for Symfony and AT LARA IOLs, respectively (*p* < 0.05). The secondary focus was measured at + 1.68 D for the Symfony IOL and at + 1.19 D for the AT LARA IOL.

Table 2 shows the MTF values recorded for each peak at 2, 3, and 4.5 m pupil sizes. The “Peak 2/1 Ratio” shows the percentage difference between the MTF values measured at the primary and secondary peaks. In all cases, the MTF value of the primary peak served as a reference value.

**Table 2:** Modulation transfer function values measured with the ISO1 and ISO2 model corneas at primary and secondary peak foci for different pupil sizes and the according Peak 2/1 Ratio percentage. *IOL = Intraocular Lens; MTF = Modulation Transfer function*

IOL	Cornea model	Pupil size	Peak 1		Peak 2		Peak 2/1 Ratio (%)
			Position in Diopter	Mean MTF value	Position in Diopter	Mean MTF value	
Symfony	ISO 1	2 mm	20	0.361	+ 1.41	0.431	119.4%
		3 mm	20	0.387	+ 1.59	0.416	107.5%
		4.5 mm	20	0.225	+ 1.85	0.252	112.0%
	ISO 2	2 mm	20	0.410	+ 1.57	0.424	103.4%
		3 mm	20	0.418	+ 1.65	0.395	94.5%
		4.5 mm	20	0.437	+ 1.69	0.344	78.7%
LARA	ISO 1	2 mm	20	0.311	+ 1.85	0.442	142.1%
		3 mm	20	0.365	+ 1.87	0.323	88.5%
		4.5 mm	20	0.487	+ 1.96	0.257	52.8%
	ISO 2	2 mm	20	0.339	+ 1.85	0.380	112.1%
		3 mm	20	0.351	+ 1.65	0.209	59.5%
		4.5 mm	20	0.169	+ 1.19	0.187	110.7%

**Fig. 2:**
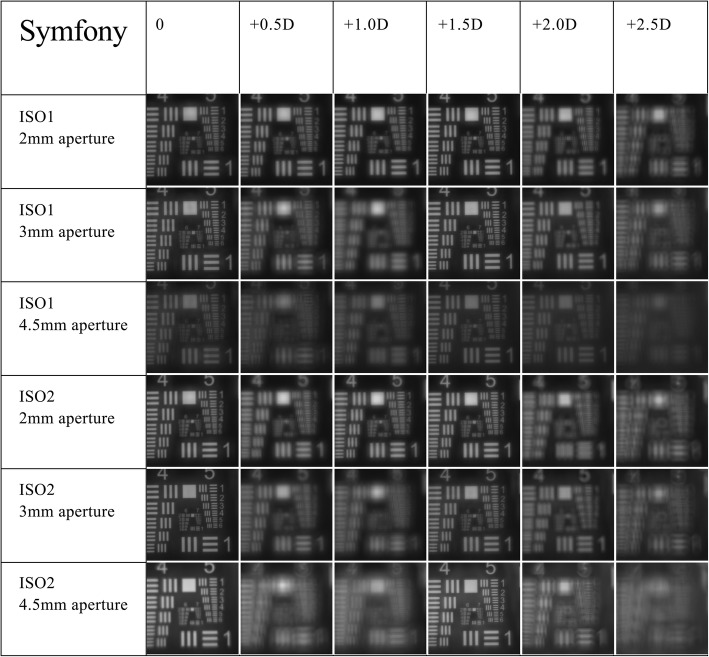
United States Air Force target images of the Symfony IOL at varying pupil sizes and defocus values with ISO1 and 2 model corneas

**Fig. 3:**
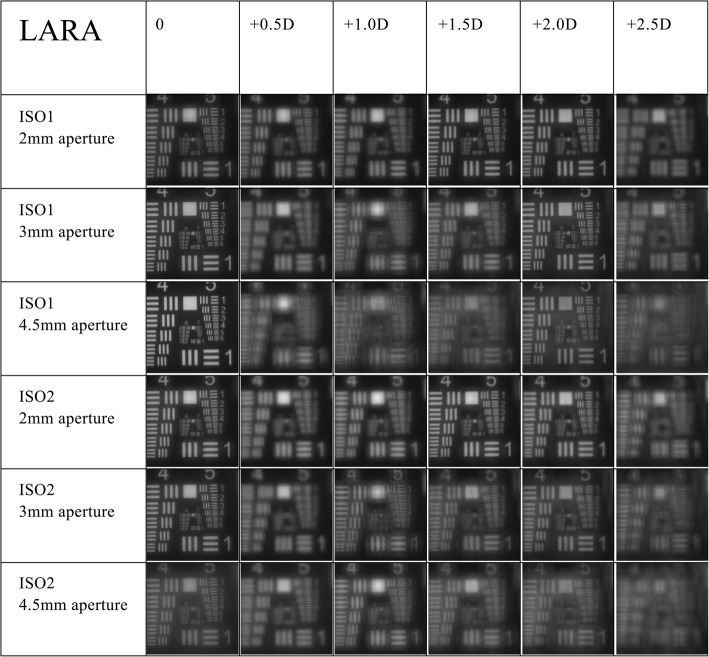
United States Air Force target images of the AT LARA IOL at varying pupil sizes and defocus values with ISO1 and 2 model corneas

The recorded USAF target images are shown in Figs. 2 and 3. The resolution of the USAF target images was generally clearer when measured with a 2 mm pupil. While the Symfony IOL demonstrated an overall better resolution with the ISO 2 model cornea (Fig. 2), the AT LARA IOL showed better image quality when measured with the ISO 1 model cornea (Fig. 3).

## Discussion

In the current laboratory study, we analyzed and compared the optical performance of two EDOF lenses, the Symfony and the AT LARA IOLs, at various pupil sizes using a standardized optical bench set-up. To our knowledge, this is the first study to conduct a head-to-head comparison of these two lenses using both ISO1 and ISO 2 model corneas.

In a previous laboratory study, Gatinel et al. evaluated the performance of a bifocal TECNIS® ZMB00 (+ 4.00 D near addition), a trifocal FineVision GFree (+ 1.75 D intermediate and + 3.50 D near additions), and the EDOF Symfony IOL, and observed that the Symfony IOL behaved as a bifocal IOL for pupil sizes of 3 and 3.75 mm, showing a V-like bimodal pattern on the through-focus MTF curve [14]. However, at 2 mm pupil size, its primary and secondary foci overlapped partially to create a broad, merged appearance, an effect the authors hypothesized to be due to the pinhole mechanism that may arise when the addition power of the secondary focus is sufficiently close to the primary one [14]. Furthermore, due to their aberration-correcting optical designs (− 0.27 μm), the bifocal and the EDOF lenses demonstrated slightly higher MTF values at all pupil sizes when measured with the ISO2 model cornea. In contrast, the low aberration-correcting trifocal lens (− 0.11 μm) showed superior performance when measured with the aberration-free ISO1 model cornea [14].

In another study, Domínguez-Vicent et al. compared the optical performance of the Symfony and the Mini WELL EDOF IOLs using an aberration-free model cornea and observed for the Symfony IOL MTF values of ca. 0.38 and 0.36 for primary and secondary foci, respectively, at 3 mm pupil size [15]. At 4.5 mm pupil size, the Symfony IOL showed a considerable reduction in its optical performance, with MTF values of ca. 0.22 and 0.23 at primary and secondary foci, respectively. Labuz et al. also compared the effects of longitudinal chromatic aberration on polychromatic image quality of different multifocal IOLs and measured for the AT LARA IOL MTF values of 0.35 and 0.26 for primary and secondary foci, respectively, at 3 mm pupil size with monochromatic green light and + 0.28 μm spherical aberration model cornea [16].

The results obtained in this study were also in agreement with the results from the aforementioned study. In most conditions, both Symfony and the AT LARA IOLs showed a bifocal-like through-focus MTF behavior. When measured with the ISO1 model cornea at 2 mm pupil size, however, the primary and the secondary foci of the two EDOF IOLs showed a merged appearance, accounting for the broad depth of focus. Overall, the Symfony IOL showed a tendency to perform better with the ISO2 model cornea at all pupil sizes, while the reverse was the case for the AT LARA IOL.

In certain aspects, the Symfony and the AT LARA IOLs share a number of similarities in their optical properties. For example, the Symfony IOL is composed of a hydrophobic acrylic material and has a refractive index of 1.47 and an Abbe number of 55. Similarly, the optical platform of the AT LARA IOL is based on its predecessors, which is made of hydrophilic-acrylic material with hydrophobic surface properties and has a refractive index of 1.46 and an Abbe number of 56.5 [9]. Furthermore, both EDOF IOLs use diffractive optical principles to effectively reduce the levels of longitudinal chromatic aberration [16, 17].

However, differences still exist between the two EDOF IOLs, particularly in respect to the spherical aberration: the Symfony IOL features a spherical aberration (SA)-correcting optic that is designed to compensate for the positive spherical aberration (SA) of the human cornea [18]. Thus, although optical aberrations are expected to increase with increasing pupil size [19], the Symfony IOL could still maintain its performance even at a pupil size of 4.5 mm. However, as was also noted by Domínguez-Vicent et al.^15^, the optical performance of this aberration-correcting IOL was lower at the same pupil size when measured with an aberration-free cornea (Fig. 1e). In contrast, the AT LARA performed better with an aberration-free cornea as its optic was designed to not induce any additional SA to the existing SA of the eye [16].

For the purpose of this study, the IOLs were tested with a monochromatic green light (wavelength of 545) as a human eye is known to be most sensitive to this wavelength [20]. It is important to note, however, that the results reported in this study merely describe the optical behavior of these IOLs in vitro, as there are other factors within the human optical pathway that may also influence the IOL’s performance in clinical situation such as light scattering resulting from the IOL material and its surface properties or visual axis misalignment. The purpose of this study, therefore, was to focus on evaluating the differences in their optical quality with regard to pupil size and spherical aberration and understanding these two factors may be helpful in selecting the appropriate IOL for each patient. Future studies should evaluate the optical performance of these IOLs with polychromatic light and different levels of tilt and decentration to simulate the real-life clinical performance.

## Conclusion

In conclusion, although the Symfony and the AT LARA EDOF IOLs share a number of similarities in their optical properties, the primary difference between the two EDOF IOLs lies in its optical behavior in relation to spherical aberration. The Symfony IOL performed better with the ISO2 cornea as it effectively compensated for the positive spherical aberration of this model cornea, while the aberration-neutral AT LARA IOL showed superior performance with the ISO1 cornea.

## Data Availability

The datasets used and/or analysed during the current study are available from the corresponding author on reasonable request.

## Abbreviations

EDOF: Extended-Depth-of-Focus
IOL: Intraocular Lens
MTF: Modulation Transfer Function
USAF: United States Air Force
